# Fish intake and the risk of brain tumor: a meta-analysis with systematic review

**DOI:** 10.1186/s12937-016-0223-4

**Published:** 2017-01-11

**Authors:** Wei Lian, Renzhi Wang, Bing Xing, Yong Yao

**Affiliations:** Department of Neurosurgery, Peking Union Medical College Hospital, Chinese Academy of Medical Science& Peking Union Medical College, Beijing, 100730 People’s Republic of China

**Keywords:** Fish intake, Nutrition, Brain tumor, Risk factors, Meta-analysis

## Abstract

**Background:**

Fish, rich in ω-3 polyunsaturated fatty acids, has been found to be associated with lower risk of several types of cancer risk, and beneficial for brain development. However, the association between fish intake and brain tumor risk is still inconsistent. Therefore, we conducted a meta-analysis to clarify the association.

**Methods:**

Relevant studies were identified from PubMed and EMBASE databases. The pooled relative risks were obtained by the fixed-effects model when no substantial heterogeneity was observed. Otherwise, the random-effects model was employed. Subgroup and publication bias analyses were also performed.

**Results:**

Nine observational studies were included in the meta-analysis. The pooled relative risk of brain cancer for the highest vs. lowest category of fish intake was 0.83 (95% confidence interval [CI]: 0.70–0.99). No significant heterogeneity was detected. Dose-response analysis showed that the RR per 100 g/day increase in fish intake was 0.95 (95% CI: 0.91–0.98). The results remained unchanged in subgroup and sensitivity analyses.

**Conclusions:**

The results of our meta-analysis suggest that fish intake might be associated with lower risk of brain cancer risk. The finding should be further confirmed by future cohort studies with validated questionnaires and strict control of confounders.

**Electronic supplementary material:**

The online version of this article (doi:10.1186/s12937-016-0223-4) contains supplementary material, which is available to authorized users.

## Introduction

Brain tumor is the neoplasmsin primary central nervous system, the incidence of brain cancer is approximately 14.4 per 100,000 persons annually, among which gliomas andmeningiomas are the two most common types, accounting for more than 80% cases [1]. Although the incidence of brain cancer is relatively low in adults, however, the prognosis of brain cancer, especially glioma, is unfavorable, thus early prevention and detection is essential to reduce the mortality of brain cancer [2, 3].

Apart from ionizing radiation and some genetic mutations, the risk factors of brain cancer are still not well understood to date [1]. Lifestyles and diet has been found to be associated with many types of cancer. As for brain cancer, N-nitroso compounds (NOC), contained in processed meat, has been long noticed to be associated with higher risk of brain tumor [4], recent meta-analysis also indicated that processed meat consumption was associated with higher risk of brain tumor [5], while intakes of vegetables, fruits [6, 7] and vitamin A [8] might reduce its risk.

As one important component of diet, fish is considered to be quite healthy, which has been found to be associated with lower risk of all-cause mortality [9], cardiovascular diseases [10] and stroke [11], as well as some forms of cancer, including colorectal [12], lung [13], prostate [14], breast [15], and liver cancers [16]. However, the association between fish intake and brain cancer risk is still inconclusive and inconsistent, and the evidence is scattered. Long-chain n-3 polyunsaturated fatty acids (PUFAs) in fish are reported to be essential for brain and intelligence development in children [17, 18]. Bunin et al. found that maternal intake of fish is associated with lower risk of subsequent brain tumors in children [19]. Moreover, recent meta-analysis showed that fish intake was associated with lower risk of Alzheimer’s disease [20]. These epidemiological and biological studies indicate the overall protective effect of fish intake on neuro system and the possibility of the inverse association between fish intake and brain tumor risk. Boeing H, el al firstly reported that there was non-significant inverse association between fish intake and brain cancer risk [21], then several following studies found that fish consumption was associated with lower risk of brain cancer significantly [22, 23]. While other studies failed to find such inverse association [24–29]. Therefore, we conducted a meta-analysis with dose-response analysis to quantify the association between fish intake and brain cancer risk.

## Materials and methods

### Search strategy

We performed and reported our meta-analysis, according to the Meta-analysis of Observational Studies in Epidemiology (MOOSE) guidelines [30]. Relevant studies were identified from PubMed and EMBASE electronic databases to Oct 2015. The following key words (or medical subject headings terms) ‘fish’ or ‘diet’ or ‘food’ or ‘nutrients’ or ‘nutrition’, in combination with ‘brain tumor’ or ‘brain cancer’ or ‘glioma’ or ‘meningioma’. No language restriction was applied in our literature search. We also checked the identified articles for more additional relevant studies.

### Inclusion criteria

Studies were included in our meta-analysis if they met the following criteria: 1) observational studies with cohort or case-control design; 2) the exposure of interest was fish intake, total fish or fresh fish intake, consumption of smoked, pickled or salted fish was not included; 3) the outcome was the risk of brain cancer (also glioma, meningioma), including both incidence and mortality; 4) relative risks[RR] (or odds rations [OR] or hazard ratios [HR]) and their corresponding 95% CIs were reported. We did not include the studies exploring the effect of fish intake on the survival of patients with brain tumor. If multiple articles were reported from the same population, then the latest one with the most information was included.

### Data extraction and quality assessment

Two authors independently reviewed the identified articles. The following information was extracted: the first author’s name, study design, location, publication year, study period, comparisons, RRs with their 95% CIs, adjusted confounding factors. The quality of each original study was also evaluated by the Newcastle-Ottawa scale, which is widely employed for assessing the observational studies [31]. Briefly, a maximum of nine points were awarded to each study on the following three sections: four for selection, three for comparability, three for exposure in case-control studies or outcome in cohort studies. In our meta-analysis, if one study scored more than six points, it would be considered as high quality. The data extraction and quality assessment were conducted independently by two authors, and discrepancies were solved with the third investigator.

### Statistical methods

RRs with their 95% CIs adjusting for most confounders in original studies were extracted for data synthesis. In some studies, RRs were reported stratified by gender or type of fish, we combined them using a fixed-effect model. Considering the differences of exposure categories in each original study, RRs comparing the highest with the lowest categories of fish intake were employed to gain a pooled estimate. Heterogeneity was evaluated using the Q and*I* ^2^ statistics [32]. For the Q statistic, *p* < 0.1 was supposed statistically significant. In this case, the random-effect model was used for pooling the RRs. Otherwise, the fixed-effects model was employed [33].

We also attempted to perform a dose-response analysis, according to the method proposed by Greenland and Longnecker [34] and Orsini et al. [35]. Studies reporting RRs with 95% CIs for at least three quantitative categories were included. We transformed all the units into g/week as a measure, and a serving of fish was assumed as 100 g, approximately. For each category, the mean or median of fish intake was assigned to each corresponding risk estimate. However, most studies included in our meta-analysis reported RRs by percentage of fish intake (e.g. tertile, quartile or quintile), in this occasion, we presumed that the average of fish intake was 300 g/week with a standard error 180 g/week, according to the state of world fisheries and aquaculture 2008 by Food and Agriculture Organization of the United Nations [36], and the midpoints of each categories were calculated, accordingly. The results of dose-response analysis were given per 100 g/week increase, and the association was also evaluated by a restricted cubic spline model with three knots. A likelihood ration test was to investigate the differences between the nonlinear and linear models [37].

In addition, we also conducted subgroup analyses by possible confounders, and meta-regression analyses to investigate the sources of heterogeneity. Influence analysis was carried out in which one individual study was removed each time, to explore the effect of each study on the overall result. Publication bias was assessed by funnel plot and Egger’s test [38], if there was an indication of significant publication bias (*P* < 0.1), we used the “trim and fill” method to correct the results [39]. All statistical analyses were performed by STATA 12.0 (StataCorp, College Satation, TX).

## Results

### Literature search and study characteristics

**Fig. 1:**
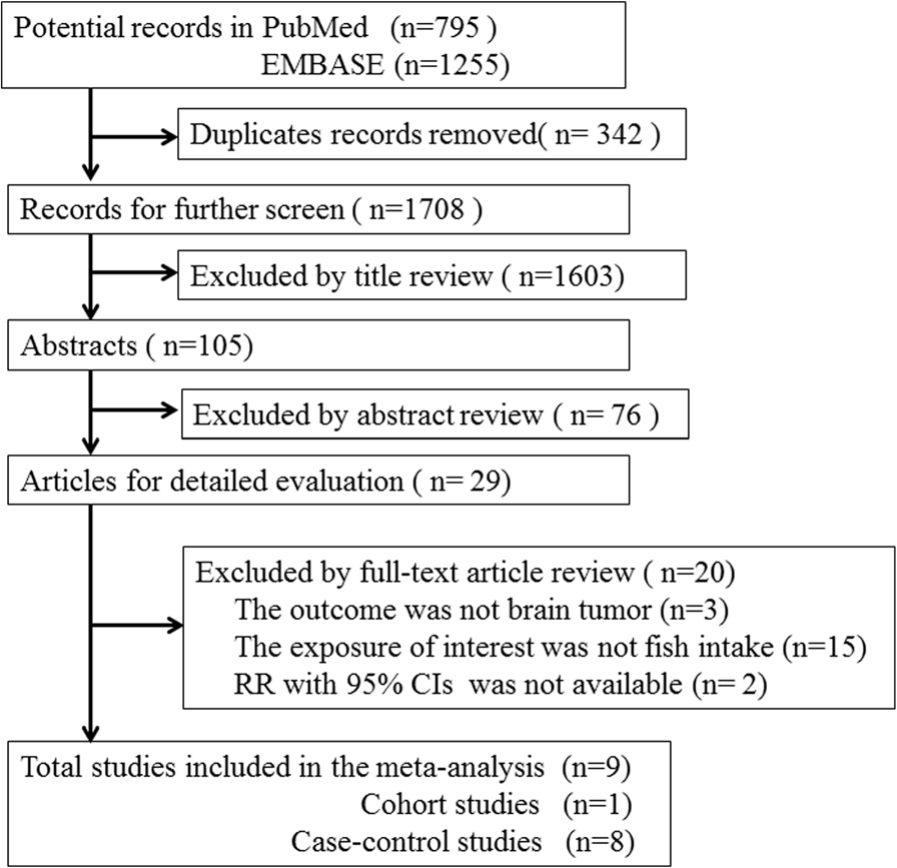
Flow chart of literature search in the meta-analysis

Originally, 1708 records were found in the electronic databases, 1679 were excluded by title and abstract review, and 29 articles were further checked by full-text, a total of nine studies were included in our meta-analysis finally. The flow diagram of literature search process was shown in Fig. 1. Among the nine studies, six studies were population-based case-control studies [21, 22, 24, 26–28], and two were hospital-based case-control studies [23, 25], one was cohort study [29]. Four studies were conducted in North America [22, 26, 27, 29]. The rest were from Germany [21], China [23], Australia [24], and Israel [25], respectively, the last one was a multi-center international study [28].

Overall, the quality scores of included studies were not quite high, with an mean of 5.78, three studies scored five points [22, 23, 25], five studies scored six points [21, 24, 26–28], only one cohort study was considered as high quality (7 points) [29]. Most studies employed food-frequency questionnaires (FFQ) (interviewed or mailed) for dietary assessment, however, only one study claimed that the FFQ was validated [29]. The most common confounding factors adjusted in the original studies were gender, age, alcohol consumption, smoking status, body mass index and total energy intake. Characteristics and overall quality scores of the included studies are summarized in Table 1.

**Table 1:** Characteristics of studies included in the meta-analysis on the association between fish intake and the risk of brain tumor

Author Year [Ref]	Duration	Design	Location	No. of case	Cohort size/contol	Dietary assessment	Pathology	Coparsions	RRs	Adjusted variables	Quality
Boeing H 1993 [21]	1987–1988	PCC	German	115	418	Questionnaire-42 items	Glioma	Tertile 1	1.00	Age, gender, tobacco-smoking and alcohol consumption	6
								Tertile 2	0.80 (0.40–1.50)		
								Tertile 3	0.70 (0.40–1.40)		
Giles GG 1994 [24]	1987–1991	PCC	Australia	409	409	Questionnaire-59 items	Glioma	Tertile 1	1.00	Alcohol and tobacco	6
								Tertile 2	1.13 (0.82–1.55)		
								Tertile 3	1.01 (0.68–1.51)		
Blowers L 1997 [22]	1986–1988	PCC	USA	94	94	Questionnaire-43 items	Glioma	Quartile 1	1.00	Total grams of food and body mass	5
								Quartile 2	0.40 (0.20–0.90)		
								Quartile 3	0.40 (0.20–1.00)		
								Quartile 4	0.40 (0.20–1.10)		
Kaplan S 1997 [25]	1987–1991	HCC	Israel	139	278	Semi-quantitative FFQ-100 items	Brain tumor	0–22 g/day	1.00	age, sex, and ethnic origin, total energy intake	5
								22.1–57 g/day	0.98 (0.60–1.39)		
								> = 57.1 g/day	0.92 (0.54–1.36)		
Hu J 1999 [23]	1993–1995	HCC	China	129	258	FFQ-57 items	Brain tumor	Quartile 1	1.00	income, education, cigarette smoking, alcohol intake, selected occupational exposures and total energy intake	5
								Quartile 2	0.80 (0.50–1.30)		
								Quartile 3	0.36 (0.20–0.80)		
								Quartile 4	0.38 (0.20–0.90)		
Chen H 2002 [26]	1988–1993	PCC	USA	236	449	FFQ-48 items	Glioma	Quartile 1	1.00	Age, age squared, gender, total energy intake, respondent type, education level, family history, and farming experience.	6
								Quartile 2	0.80 (0.50–1.30)		
								Quartile 3	0.70 (0.40–1.10)		
								Quartile 4	0.60 (0.30–1.20)		
Hu J 2008 [27]	1994–1997	PCC	Canada	1009	5039	FFQ-69 items	Brain tumor	Tertile 1	1.00	age group, province, education, body mass index, sex, alcohol use, pack-year smoking, total of vegetable and fruit intake, and total energy intake.	6
								Tertile 2	0.90 (0.70–1.10)		
								Tertile 3	0.80 (0.60–1.00)		
Terry MB 2009 [28]	1984–1991	PCC	Europe, Northern American and Australia	1548	2486	Dietary questionnaire	Brain tumor	Quartile 1	1.00	Age, sex, center and the following food groups: leafy green vegetables, yellow–orange vegetables, cured meat, non-cured meat, fresh fish, dairy eggs, grains, and citrus fruit	6
								Quartile 2	0.90 (0.80–1.10)		
								Quartile 3	0.90 (0.70–1.10)		
								Quartile 4	0.90 (0.70–1.10)		
Daniel CR 2011 [29]	1995–2006	Cohort	USA	749	492,186	FFQ-124 items	Brain tumor	Quintile 1	1.00	red meat intake, age, sex, education, marital status, family history of cancer, race, BMI, smoking status, frequency of vigorous physical activity, MHT in women, and intake of alcohol, fruit, vegetables, and total energy	7
								Quintile 2	1.14 (0.91–1.45)		
								Quintile 3	1.04 (0.82–1.33)		
								Quintile 4	1.22 (0.97–1.55)		
								Quintile 5	1.05 (0.83–1.35)		

*Abbreviations*: *FFQ* food frequency questionnaire, *PCC* population-based case-control study, *HCC* hospital-based case-control study, *BMI* body mass index, *MHT* menopausal hormone therapy

### Overall analysis

**Fig. 2:**
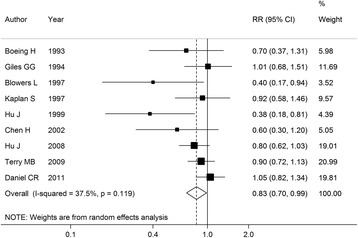
Forest plot of fish intake and brain tumor risk for high versus low consumption. RR, relative risk; CI, confidence interval

A moderate heterogeneity was observed among studies, with *I* ^2^ = 37.5%, *P* = 0.12. Thus, a random-effects model was used to combine the RRs, we observed a significant inverse association between fish intake and the risk of brain tumor, with a pooled RR of 0.83 (95% CI: 0.70–0.99), as shown in Fig. 2.

### Dose-response analysis

**Fig. 3:**
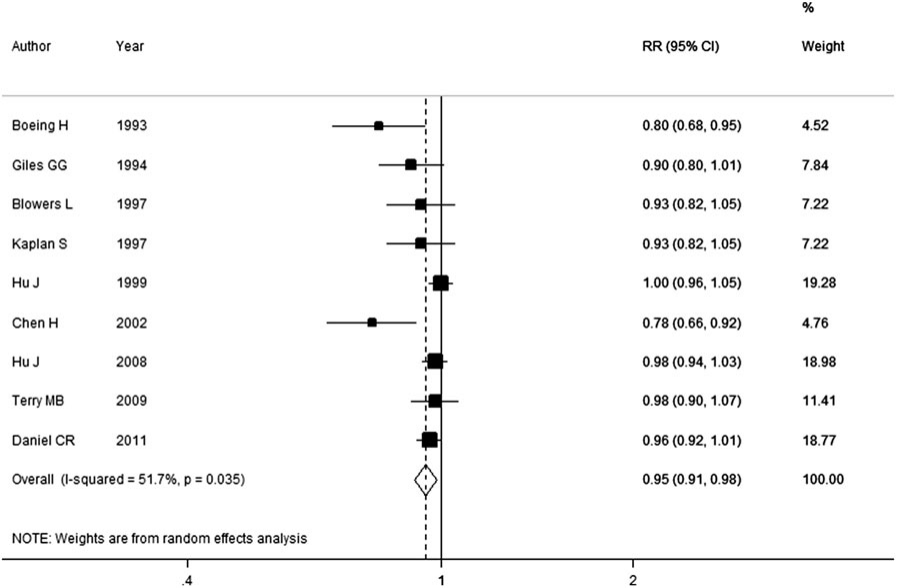
Forest plot of fish intake and brain tumor risk per 100 g/week increase. RR, relative risk; CI, confidence interval

**Fig. 4:**
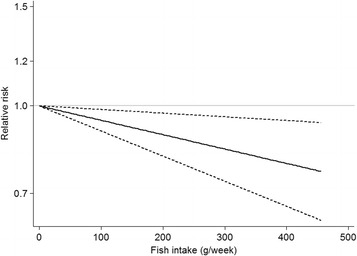
Dose-response analysis for fish intake and brain tumor risk. The P value for non-linear test was 0.85, and a linear model was used. *Black solidline* represents the trend between fish intake and brain tumor risk, and *short dashed lines* represent the 95% confidence intervals

All the original studies reported the results for at least three quantitative levels of fish intake, and were included into the dose-response analysis. The results showed that the summary RR per 100 g/week increment in fish intake was 0.95 (95% CI: 0.91–0.98), with an evident heterogeneity (*I* ^2^ = 51.7%; P_*heterogeneity*_ = 0.04) (Fig. 3). The *P* value for non-linear test was 0.85 (*P* > 0.1), so a linear model was used to evaluate the dose-response relationship between fish intake and brain tumor risk, as shown in Fig. 4, the inverse association was also detected.

### Subgroup analyses

In subgroup analyses stratified by study design, the pooled RR for five population-based case-control studies was 0.83 (95% CI: 0.72–0.96) using a fixed-effects model, with little heterogeneity (*I* ^2^ = 8.2%; P_*heterogeneity*_ = 0.36), and the RR for two hospital-based case-control studies was 0.62 (95% CI: 0.26–1.47), with significant heterogeneity (*I* ^2^ = 74.1%; P_*heterogeneity*_ = 0.05), however, only one cohort study was included in the meta-analysis, and the result was non-significant [29].

After stratifying by pathology types, five studies [21, 22, 24, 26, 28] reported results between fish intake and the risk of glioma, a pooled analysis yielded an RR of 0.81 (95% CI: 0.64–1.03), with a low heterogeneity (*I* ^2^ = 24.6%, P_heterogeneity_ = 0.26). The results for the association between meningioma and fish intake were always presented in combination with other brain tumors and ineligible for subgroup analysis. Besides, Terry MB et al. [28] reported an protective effect of fish intake on oligodendroglioma in a multi-center international study.

### Meta-regression, influence analyses and Publication bias

Meta-regression analyses were performed to explore the sources of heterogeneity, however, none of the confounding factors were found to be significantly associated with heterogeneity, including publication year (*P* = 0.74), study design (*P* = 0.26) location (*P* = 0.39) and number of cases (*P* = 0.75). After removing the study by Hu J et al. in 1999 year [23], the heterogeneity among studies was reduced significantly, from *I* ^2^ of 51.7% to 13.0%. Influence analysis by omitting one study each time showed that the overall results were not changed significantly, ranging from 0.79 (95% CI: 0.64–0.99) to 0.88 (95% CI: 0.77–1.00).

**Fig. 5:**
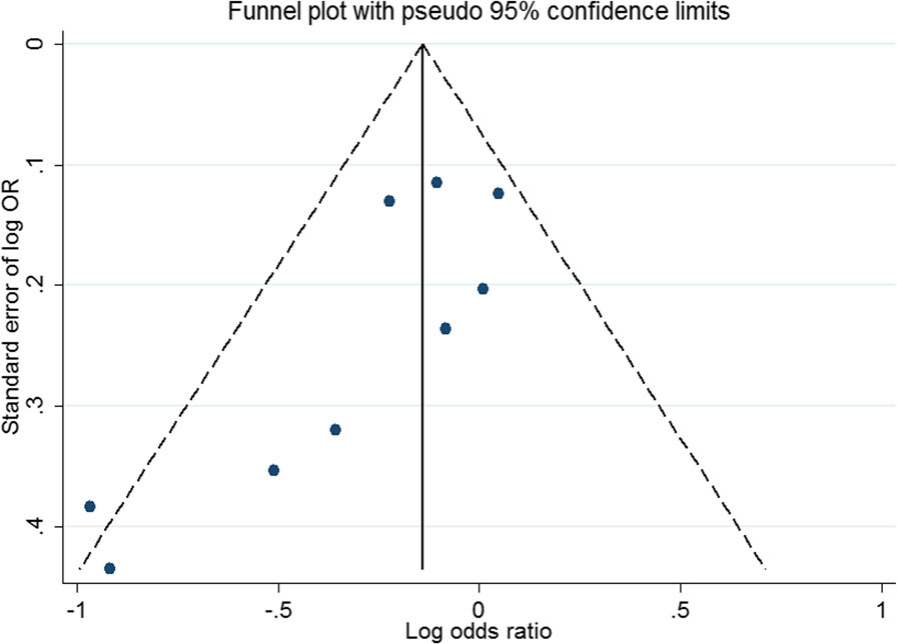
Funnel plot for all studies included in the meta-analysis

The funnel plot seemed to a little unsymmetrical (Fig. 5), and Egger’s test also indicated the existence of publication bias (*P* = 0.02). The “trim and fill” method was used to correct such bias, however, no studies were found to be filled, and data remained unaltered, implying that the effect of publication bias on the overall results might be tiny.

## Discussion

To our best knowledge, this is the first meta-analysis with systematic review on the association between fish intake and brain cancer risk. Based on nine observational studies, involving a total of 4428 brain cancer cases and 501,617 participants, the results showed that fish intake might be associated with lower brain cancer risk, and dose-response analysis also supported the conclusion. However, due to the small number of studies included, the statistical power was weakened to some extent, and further subgroup analyses were limited.

Although the precise mechanisms whereby fish intake reduces the risk of brain cancer are still unknown, the biological explanation is plausible. Fish is rich in polyunsaturated fatty acids (PUFAs), high quality proteins and minerals, and low in fat and cholesterol, especially, marine fish contains plenty of omega-3 fatty acids, including docosahexaenoic acid (DHA) and eicosapentaenoic acid (EPA), which exert a series of health benefits and neuroprotective properties [40, 41]. Studies in vitro or in animal models have shown that PUFAs exhibit anti-tumor effect through multiple mechanisms, including inducing apoptosis, growth inhibition, anti-angiogenesis and enhancement of immune system [42, 43]. Several studies in vivo or animal models also showed that PUFAs might induce cell cycle arrest, apoptosis-specific expression changes and enhance anticancer properties of lomustine in brain tumor cells [44–46]. All of these findings suggest a potential protective role of fish on brain cancer development.

Notably, our analysis is focused on total fish intake, including fresh and frozen fish, however, smoked, pickled or salted fish was not included in our study, further literature review found that five studies [21–24, 47] involved data on the association between smoked fish or salted fish intake and the risk of brain tumor, and most of them found a non-significant positive relationship between them, the pooled analysis yielded an overall RR of 2.41 (95% CI: 1.01–5.75) (Additional file 1 Figure S1), indicating that processed fish might be associated with increased brain cancer risk. Accordingly, a recent meta-analysis also found that processed meat might increase the risk of brain cancer [5]. The different effects of fresh and processed fish might be caused by the carcinogens induced by food storage and processing. Therefore, people are encouraged to eat more fresh fish, however, processed fish, including smoked, pickled or salted fish should be avoided.

The strength of our study lies in a large sample size (4428 brain cancer cases and 501,617 participants) and long duration (from 1986 to 2006), thus to enhance the statistical power to detect possible weak association. Moreover, dose-response analysis was conducted to quantify the association. However, several shortcomings must be also acknowledged in our study when interpreting the results. Firstly, the original studies included were mostly low-quality case-control studies, which were prone to recall and selection bias, and there was only one prospective cohort study with a null finding between fish intake and brain tumor risk. Secondly, unvalidated questionnaires and inadequate adjustment for potential confounders, including irradiation exposure, total energy intake and body mass index, would also influence the stability of the results. Thirdly, characteristics of study population, sample size, follow-up duration, and exposure levels of fish intake were different across the original studies, which might contribute the heterogeneity among studies and lead to incomparability of results, however, both highest category of fish intake vs. the lowest analysis and dose-response analysis were conducted and the results were consistent. Besides, since the number of included studies was small, subgroup analyses by common confounders including location, design, publication year and types of fish were not performed. Lastly, we only searched publications in English, possible publication bias was inevitable. Indeed, there was an indication of publication bias with Egger’s test. However, the statistical power of the test was relatively low due to the small number of studies included, and no studies were added when employing the “trim and fill” method, suggesting that the influence of publication bias on results was insignificant.

## Conclusions

In summary, the present meta-analysis suggests that fish intake is associated with lower risk of brain tumor risk. Given the small number of included studies, the finding should be further confirmed by further prospective cohort studies with validated questionnaires and strict control of confounders.

## Additional file

Additional file 1: Figure S1. Forest plot of processed fish intake and brain tumor for high versus low consumption. RR, relative risk; CI, confidence interval. (TIF 1551 kb)

## Abbreviations

CI: Confidence interval
DHA: Docosahexaenoic acid
EPA: Eicosapentaenoic acid
HR: Hazard ratios
MOOSE: Meta-analysis of Observational Studies in Epidemiology
NOC: N-nitroso compounds
OR: Odds rations
RR: Relative risk
