# Supplementary figures and images for: Fish intake and the risk of brain tumor: a meta-analysis with systematic review
Source: Nutr J. 2017 Jan 11;16:1. doi: 10.1186/s12937-016-0223-4 (PMC5225583; doi:10.1186/s12937-016-0223-4)

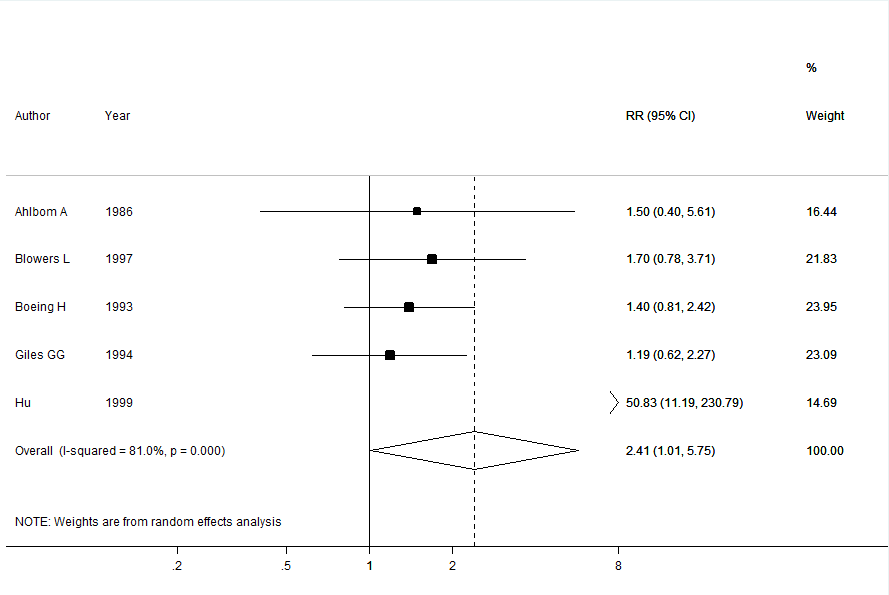

Supplement: Additional file 1: Figure S1. — Forest plot of processed fish intake and brain tumor for high versus low consumption. RR, relative risk; CI, confidence interval. (TIF 1551 kb) [file 12937_2016_223_MOESM1_ESM.tif]
